# Something Old Brings New Insights: Activity of Cerivastatin Against Thermally Dimorphic Fungi and Its Potential as an Antifungal Scaffold

**DOI:** 10.3390/ph19030479

**Published:** 2026-03-14

**Authors:** Luana P. Borba-Santos, Sonia Rozental

**Affiliations:** Laboratório de Biologia Celular de Fungos, Centro de Pesquisa em Medicina de Precisão, Instituto de Biofísica Carlos Chagas Filho, Universidade Federal do Rio de Janeiro, Rio de Janeiro 21941-599, Brazil; rozental@biof.ufrj.br

**Keywords:** *Sporothrix brasiliensis*, *Sporothrix schenckii*, *Sporothrix globosa*, sporotrichosis, NIH Clinical Collection library

## Abstract

**Background/Objectives**: Species of the *Sporothrix* genus were used as a biological model to identify potential antifungal compounds within the NIH Clinical Collection library. **Methods**: A total of 707 compounds were screened for antifungal activity using in vitro susceptibility assays. Compounds exhibiting significant inhibitory effects (growth inhibitions greater than 80%) were further evaluated by determining their minimum inhibitory concentrations (MICs). As cerivastatin demonstrated the highest activity after itraconazole, it was selected for further evaluation, either alone or in combination with itraconazole, using susceptibility assays, electron microscopy, and flow cytometry analyses. **Results**: Among the screened compounds, twenty-six showed significant inhibition of yeast growth (≥80%). Compounds with previously reported antifungal activity or not used as oral treatment were excluded from further analysis. MIC determination of eleven selected compounds revealed that cerivastatin inhibited the growth of *Sporothrix brasiliensis*, *Sporothrix schenckii*, and *Sporothrix globosa* at concentrations of 1.25 µM and 0.6 µM. Combination treatment with cerivastatin and itraconazole resulted in greater inhibition of *Sporothrix* growth than either agent alone. Flow cytometry and microscopic analyses revealed more pronounced morphophysiological alterations in yeast cells following combination treatment. **Conclusions**: These findings highlight the potential of cerivastatin as an antifungal agent when used in combination with itraconazole. Furthermore, the chemical scaffold of cerivastatin warrants further investigation as a basis for the development of novel statins with antifungal activity.

## 1. Introduction

Fungal infections are one of the leading causes of infectious disease-related deaths worldwide [1,2]. The main antifungal classes used to treat these infections are limited by suboptimal efficacy, toxicity, drug interactions, and poor activity against resistant species [3]. Therefore, the study of more effective compounds that act more selectively on fungal cells and require shorter treatment times is essential.

The evaluation of libraries containing hundreds of molecules is a useful tool for discovering promising chemical groups with antifungal activity [4]. This approach enables the study of a wide range of molecules with diverse physicochemical characteristics, which can serve as a good scaffold for the development of new antifungals.

In the present work, we used pathogenic species of the *Sporothrix* genus as a biological model for medically relevant fungi: *Sporothrix brasiliensis*, *Sporothrix schenckii*, and *Sporothrix globosa*. These species cause sporotrichosis, a neglected tropical disease that is the most common subcutaneous mycosis worldwide [5,6]. The main sporotricosis manifestation is lymphocutaneous disease, followed by cutaneous form; however, sporotrichosis can assume ocular, pulmonary, osteoarticular, meningitis, and other manifestations [5].

*Sporothrix* spp. are thermodimorphic fungi, present in filamentous form in nature (28 °C) and or in yeast form when infected the mammalian host (37 °C). The most virulent species is *S. brasiliensis* [5], which is also the most frequent species observed in Brazil. Sporotrichosis is hyperendemic in various regions of the country, with zoonotic transmission by infected cats being the primary mode of disease acquisition [5].

Reports of therapeutic failures and the identification of isolates with reduced sensitivity to itraconazole (the reference antifungal used to treat sporotrichosis) have become more frequent in recent years [5]. Here, we evaluated the NIH Clinical Collection library against *Sporothrix* yeasts to discover promising chemical groups with antifungal activity, and studied the effects of the most promising compound combined with itraconazole. We showed that cerivastatin inhibited *Sporothrix* growth and enhanced itraconazole activity.

## 2. Results

### 2.1. NIH Clinical Collection Screening Reveals Antifungal Activity of Cerivastatin

Initially, the in vitro susceptibility of *S. brasiliensis* yeasts cells to compounds from the NIH Clinical Collection library was evaluated at a concentration of 10 µM. Among the 707 molecules tested, 26 inhibited more than 80% of yeast growth (Table 1 and Table S1). From these group, fifteen compounds with previously reported antifungal activity as well as compounds with previously antifungal descriptions and not used as oral treatment were excluded from further analysis [5,6,7,8].

Minimum inhibitory concentrations (MICs) were determined using the broth microdilution method for the remaining eleven compounds against yeast forms of *Sporothrix brasiliensis*, *Sporothrix schenckii*, and *Sporothrix globosa* (Table 2). Itraconazole was included as the reference antifungal agent. Among these eleven compounds, cerivastatin exhibited the most potent antifungal activity after itraconazole, inhibiting the growth of all three *Sporothrix* species at concentrations as low as 1.25 µM (Table 2).

In addition to cerivastatin, other statins present in the NIH Clinical Collection library (fluvastatin, pitavastatin, lovastatin, mevastatin, pravastatin, and simvastatin) were also evaluated in the growth inhibition assay. In contrast to cerivastatin, which induced marked growth inhibition, none of the other statins exhibited significant inhibitory activity against *Sporothrix brasiliensis* at a concentration of 10 µM (Table 3).

### 2.2. Cerivastatin Enhances the Anti-Sporothrix Activity of Itraconazole

**Figure 1:**
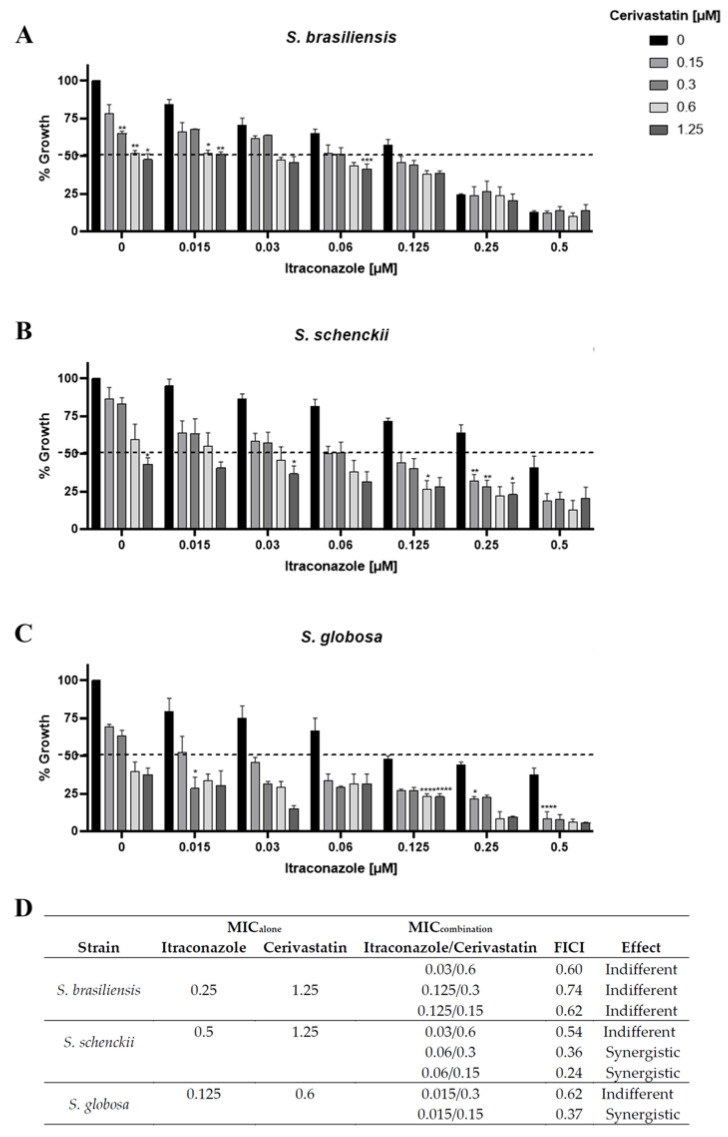
In vitro evaluation of the combined activity of cerivastatin and itraconazole after 48 h of treatment. (**A**–**C**) with *S. brasiliensis* (**A**), *S. schenckii* (**B**), and *S. globosa* (**C**). (**D**) Most effective combinations of cerivastatin and itraconazole against *Sporothrix* species based on the fractional inhibitory concentration index (FICI) values. * *p* < 0.05, ** *p* < 0.01, *** *p* < 0.001, **** *p* < 0.0001 vs. treatment with itraconazole only, at the same concentration used in the combination test (by one-way ANOVA with Dunnett’s test).

Cerivastatin, like other statins, inhibits the enzyme HMG-CoA reductase (3-hydroxy-3-methylglutaryl-coenzyme A reductase), a key component of the sterol biosynthesis pathway [11]. Itraconazole also targets sterol biosynthesis but acts at a distinct step by inhibiting C14-α-lanosterol demethylase. To evaluate the potential synergistic interaction between cerivastatin and itraconazole, a checkerboard assay was performed. The results indicated that the combination of the two compounds produced a greater inhibitory of *Sporothrix* growth than either agent alone (Figure 1A–C). Analysis of the fractional inhibitory concentration index (FICI) revealed synergistic interactions for *S. schenckii* and *S. globosa* (FICI ≤ 0.5) when the drugs were combined (Figure 1D). In *S. brasiliensis*, lower concentrations of the combined drugs were effective to inhibit fungal growth, reducing MIC values by more than twofold; however, no synergistic effect was observed according to the FICI criteria (Figure 1D).

**Figure 2:**
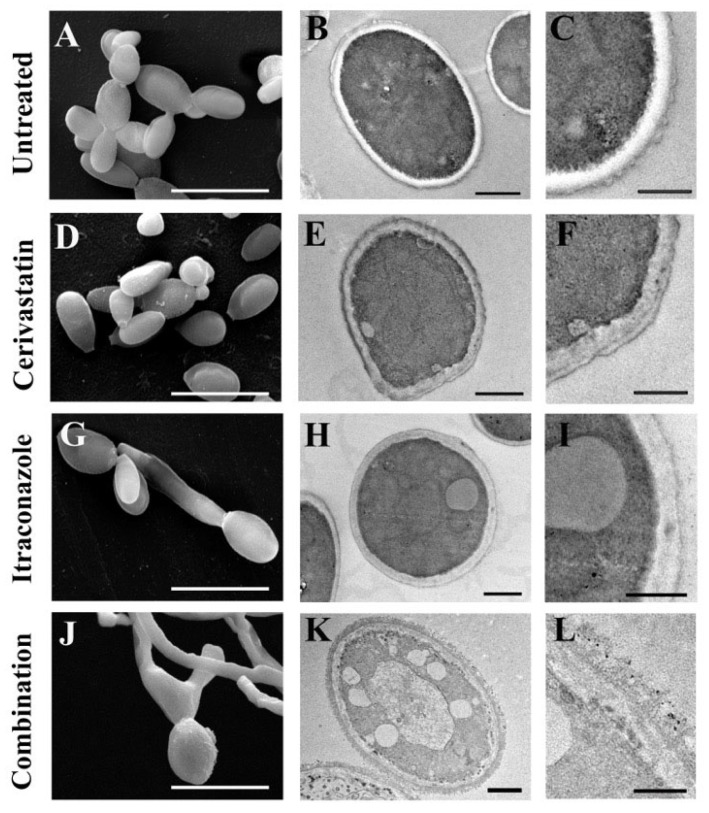
Ultrastructural analysis of *Sporothrix brasiliensis* cells after exposure to cerivastatin and itraconazole for 48 h. Scanning electron microscopy (**A**,**D**,**G**,**J**) and transmission electron microscopy (**B**,**C**,**E**,**F**,**H**,**I**,**K**,**L**) revealed that the treatments induced the formation of electron-lucent vacuoles within the cytoplasm, as well as alterations in the plasma membrane and cell wall. These ultrastructural changes were markedly more pronounced following exposure to the combination treatment. Scale bars: 10 µm (**A**,**D**,**G**,**J**); 0.5 µm (**B**,**E**,**H**,**K**); 0.25 µm (**C**,**F**,**I**,**L**).

To compare the cellular effects of cerivastatin in combination with itraconazole, *S. brasiliensis* cells were treated with 0.125 µM itraconazole and 0.15 µM cerivastatin for 48 h and subsequently analyzed by scanning electron microscopy (SEM), transmission electron microscopy (TEM), and flow cytometry. SEM analysis showed that untreated cells displayed an elongated morphology, which was also observed in cells treated with cerivastatin alone (Figure 2A,C). In contrast, yeast-to-hyphae conversion was observed following exposure to itraconazole and to the combination treatment (Figure 2E,G). TEM analysis of untreated cells revealed an electron-dense cytoplasm surrounded by an intact cell wall and plasma membrane (Figure 2B). Treatment with either cerivastatin or itraconazole led to the formation of electron-lucent vacuoles within the cytoplasm, with cerivastatin additionally inducing marked alterations in the cell wall and plasma membrane (Figure 2D,F). Cells exposed to the combination therapy exhibited extensive cytoplasmic disorganization, pronounced accumulation of electron-lucent vacuoles, fragmentation of the plasma membrane, and severe structural disruption of the cell wall (Figure 2H).

**Figure 3:**
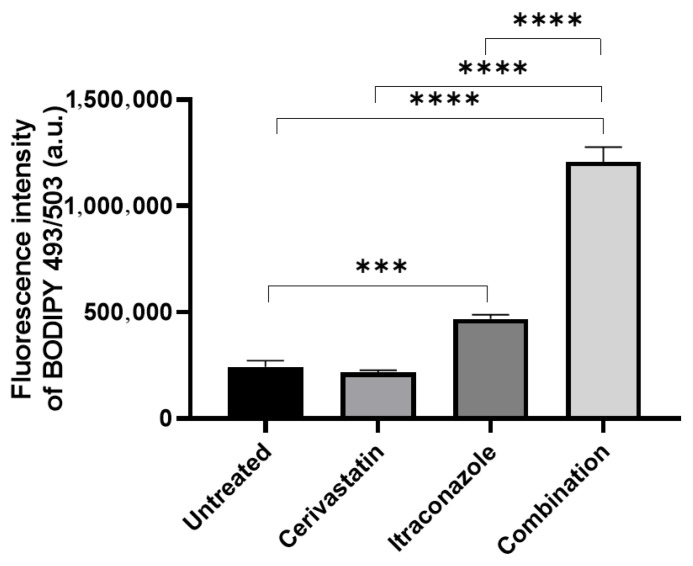
Effect of cerivastatin and itraconazole combination on the levels of neutral lipids in *Sporothrix brasiliensis*. After 48 h of exposure, yeast cells were stained with BODIPY 493/503 and analyzed by flow cytometry. The combination of cerivastatin and itraconazole resulted in a marked increase in neutral lipid levels compared with the untreated control and with either drug alone. Data are presented as mean ± SEM from a representative experiment. *** *p* < 0.001, **** *p* < 0.0001 (by one-way ANOVA with Bonferroni’s test).

We investigated whether treatment with the combination of cerivastatin and itraconazole increased neutral lipid accumulation using the fluorescent probe BODIPY 496/503 to stain fungal cells (Figure 3). Cerivastatin alone did not induce neutral lipid accumulation, whereas itraconazole treatment resulted in a statistically significant increase in neutral lipid levels. Notably, this effect was further amplified when the two drugs were used in combination (Figure 3). Taken together, these findings indicate that the combined treatment with cerivastatin and itraconazole induces more pronounced morphophysiological alterations in fungal cells than either drug alone.

## 3. Discussion

The limitations of current therapies for fungal infections highlight the need to explore alternative molecular scaffolds and cellular targets. In this study, we screened 707 compounds from the NIH Clinical Collection library, which contains molecules with a documented history of use in human clinical trials, to identify those with significant antifungal activity against pathogenic species of the genus *Sporothrix*. Our results identify cerivastatin as the most promising candidate in this group.

Cerivastatin stood out for its ability to inhibit the growth of three clinically relevant *Sporothrix* species (*S. brasiliensis*, *S. schenckii*, and *S. globosa*) at low concentrations (<1.25 µM). Moreover, cerivastatin exhibited synergistic activity when combined with itraconazole, the first-line antifungal therapy for sporotrichosis. Cerivastatin is a statin originally developed in the 1990s to treat hyperlipidemia and prevent cardiovascular disease, and was withdrawn from the market in 2001 due to an increased incidence of rhabdomyolysis [12]. Curiously, other statins evaluated in this study did not display a significant inhibition of fungal growth (Table 3). The distinctive structural features of cerivastatin may account for its enhanced affinity for fungal HMG-CoA reductase (3-hydroxy-3-methylglutaryl-coenzyme A reductase), resulting in a stronger inhibitory effect on fungal growth compared with other statins. HMG-CoA reductase is the primary target of statins and plays a central role in metabolic pathways responsible for the synthesis of essential cellular components, particularly sterols, cholesterol in humans, and ergosterol in fungal cells [11,13].

Statins are widely prescribed as first-line agents for the control of hypercholesterolemia and prevention of cardiovascular disease [14]. In addition to their lipid-lowering properties, particularly the reduction in LDL cholesterol, these drugs have been increasingly investigated for pleiotropic effects, including anti-inflammatory and immunoregulatory activities [14]. Moreover, accumulating evidence indicates that statins possess diverse biological actions, with reported antimicrobial effects that extend to antiparasitic, antibacterial, antiviral, and antifungal activity [14]. The antifungal potential of statins appears to be multifactorial. One of the primary mechanisms involves the disruption of sterol biosynthesis in fungi through the inhibition of HMG-CoA reductase, a key enzyme in the mevalonate pathway. This interference not only compromises ergosterol production but also alters other processes such as protein prenylation, mitochondrial integrity, and cellular signaling pathways, which may culminate in apoptotic cell death. Additionally, statins have been associated with disturbances in fungal morphogenesis and cell cycle progression [13]. By targeting HMG-CoA reductase, these compounds decrease the synthesis of mevalonate-derived intermediates, including farnesyl pyrophosphate. The resulting impairment of isoprenoid formation limits protein isoprenylation, a post-translational modification essential for the proper localization and function of regulatory proteins [13]. Consequently, processes governing cell proliferation, differentiation, programmed cell death, respiration, and iron homeostasis may be adversely affected in fungal cells [13].

The antifungal activity of cerivastatin has been previously demonstrated against filamentous fungi, with minimum inhibitory concentrations (MICs) ranging from 0.04 to 0.14 µM against *Trichophyton* species, 0.03 µM against *Aspergillus fumigatus*, and 1.61 µM and 0.4 µM against the hyphal and conidial forms of *Exserohilum rostratum*, respectively [15,16,17]. In contrast, the in vitro inhibitory activity of other statins, including lovastatin, simvastatin, pravastatin, and fluvastatin, has been reported against a broad range of pathogenic fungi, such as species of *Candida*, *Cryptococcus*, *Aspergillus*, dermatophytes, and mucormycetes [13,18]. However, antifungal effects are generally observed only at relatively high drug concentrations [13,18], which may explain the limited activity of these statins when tested at 10 µM against *S. brasiliensis* in the present study.

Cholesterol is the predominant sterol in the plasma membrane of mammalian cells, whereas ergosterol is the principal sterol in the plasma membrane of most pathogenic fungi [11,13]. Inhibition of ergosterol biosynthesis represents one of the main mechanisms exploited by clinically available antifungal agents. The azole class, the most widely used antifungals, targets C14-α-lanosterol demethylase, an enzyme that catalyzes a later step in sterol biosynthesis than HMG-CoA reductase [11,13]. Disruption of ergosterol synthesis compromises membrane integrity, fluidity, and permeability, thereby increasing fungal susceptibility to osmotic, oxidative, and cell wall stresses. Moreover, the accumulation of alternative sterol intermediates can exert toxic effects on fungal cells [11,13,19].

Targeting distinct steps of the sterol biosynthesis pathway through drug combinations represents a promising antifungal strategy. Consistent with previous reports showing that statins can potentiate the activity of azoles against diverse fungal pathogens [13], we investigated whether cerivastatin could exert synergistic effects in vitro when combined with itraconazole against *Sporothrix* species, and demonstrated that cerivastatin significantly enhanced the antifungal activity of itraconazole against *Sporothrix* yeasts (Figure 1).

Based on these findings, we selected the lowest concentration of cerivastatin that produced significant growth inhibition in combination with itraconazole against *S. brasiliensis* yeasts to further assess the morphophysiological changes. *S. brasiliensis* was chosen due to its major epidemiological relevance in Brazil [5]. Scanning and transmission electron microscopy revealed profound ultrastructural alterations after the combined treatment, indicating that simultaneous inhibition of two distinct steps in the ergosterol biosynthesis pathway markedly increased fungal cell damage (Figure 2).

Inhibition of ergosterol biosynthesis can result in the intracellular accumulation of neutral lipids, which correspond to sterol intermediates generated during incomplete ergosterol synthesis [11]. Flow cytometric analysis revealed significantly higher levels of neutral lipid accumulation following treatment with the cerivastatin–itraconazole combination compared with either cerivastatin or itraconazole alone (Figure 3). These findings support the notion that the enhanced antifungal effect of the combined treatment is primarily associated with the intensified inhibition of ergosterol biosynthesis.

In recent years, several studies have highlighted the potential of statins as antifungal agents when repurposed in combination with azoles [13]. Nevertheless, additional in vivo studies and clinical evidence are still required to substantiate their therapeutic applicability. From this perspective, accumulating data on the antifungal activity of statins suggest that their chemical scaffolds could be exploited for the development of compounds with increased selectivity toward fungal cells. Notably, comparative sequence analysis indicates limited similarity between fungal and human HMG-CoA reductase (approximately 55% identity), as determined by BLASTp alignment of the human and *S. brasiliensis* enzymes (XP_011541659.1 and XP_040622855.1, respectively) [20]. This divergence may provide a molecular basis for the rational design of fungal-selective inhibitors.

It is important to emphasize that the other compounds that demonstrated anti-*Sporothrix* activity and were not explored in this study are potential subjects for future investigations, including studies involving other pathogenic fungi. In the present work, only the compounds capable of markedly inhibiting *S. brasiliensis* at 10 micromolar, whose antifungal activity had not been previously described and which are used in oral treatments, were selected for the determination of inhibitory concentrations, with cerivastatin being the most promising compound.

In conclusion, our findings highlight the potential of cerivastatin, particularly in combination with itraconazole, as a promising strategy against agents of sporotrichosis. Moreover, the chemical structure of cerivastatin represents a valuable scaffold for the development of novel statins with antifungal properties. We anticipate that the insights generated by this study will contribute to the identification of new therapeutic targets and foster the development of more effective antifungal agents.

## 4. Materials and Methods

### 4.1. Fungal Strains and Growth Conditions

Three reference isolates, *S. brasiliensis* ATCC MYA 4823, *S. schenckii* ATCC 32286, and *S. globosa* CBS 130104, were used in this study. To obtain filamentous growth, strains were maintained on Sabouraud agar (Kasvi, São José dos Pinhais, PR, Brazil) at 25 °C for seven days. Thermal dimorphism was subsequently induced by transferring the cultures to brain heart infusion agar (BD Difco, Franklin Lakes, NJ, USA) and incubating them for an additional seven-day period at 35 °C in an atmosphere containing 5% CO_2_. After this phase transition, cells in the yeast (parasitic) form were collected and used throughout all experimental procedures.

### 4.2. Chemical Library and Reference Drug

A collection of clinically characterized small molecules from the NIH Clinical Collection, provided by the National Center for Advancing Translational Sciences, was screened in this work. Each compound was solubilized in dimethyl sulfoxide (DMSO) to generate 10 mM stock solutions, which were stored at −20 °C until required. Itraconazole (1 mM; Sigma-Aldrich^®^, MO, USA) prepared in DMSO was included as a positive antifungal control.

### 4.3. Screening of the NIH Clinical Collection Library

To identify molecules with antifungal activity, *S. brasiliensis* ATCC MYA 4823 was selected as the screening model. Test compounds were prepared at 10 μM in RPMI 1640 medium (Sigma-Aldrich^®^, St. Louis, MO, USA) supplemented with 2% glucose and buffered with 0.165 M MOPS (pH 7.2). Aliquots were dispensed into flat-bottom 96-well microplates (Kasvi, São José dos Pinhais, PR, Brazil). Yeast cells were then inoculated at a final concentration of 1 × 10^5^ CFU/mL per well, and plates were incubated for 48 h at 35 °C in a 5% CO_2_ environment. After incubation, fungal proliferation was qualitatively examined under an inverted microscope and quantitatively determined by optical density measurement at 492 nm. Absorbance readings were corrected by subtracting background values obtained from wells containing only supplemented RPMI. Growth inhibition percentages were calculated relative to the untreated controls according to the equation: I = 100 − (A × 100/C), where A represents treated samples and C corresponds to untreated controls. Wells containing 0.1% DMSO were included to exclude solvent-related effects. Compounds producing more than 80% inhibition were selected as hits, consistent with marked suppression of visible growth. The screening data reflect two independent assays performed with four technical replicates each.

### 4.4. Determination of Minimum Inhibitory Concentration

The antifungal potency of selected hits was further characterized by determining the minimum inhibitory concentrations using a broth microdilution protocol adapted for *Sporothrix* spp. [21]. Compounds were serially diluted in supplemented RPMI to yield final concentrations between 0.02 and 10 μM in 96-well plates. Yeast suspensions were added to achieve a final density of 1 × 10^5^ CFU/mL and incubated under the same temperature and CO_2_ conditions described above. Post-incubation assessment included microscopic evaluation and absorbance measurement at 492 nm. The MIC was defined as the lowest drug concentration resulting in at least 50% inhibition of fungal growth, determined using the same inhibition formula described above. Values are reported as the average of three independent experiments conducted in duplicate.

### 4.5. Evaluation of the Interaction Between Cerivastatin and Itraconazole

Itraconazole–cerivastatin interactions were investigated using a checkerboard microdilution assay as previously described [22]. *S. brasiliensis* yeast suspensions (1 × 10^5^ CFU/mL) were distributed into microplates containing serial dilutions of cerivastatin (0.15–10 μM) along one axis and itraconazole (0.001–1 μM) along the other. Following incubation for 48 h at 35 °C under 5% CO_2_, fungal growth was assessed and minimum inhibitory concentrations (MICs) were established for each compound alone and in combination. The interaction profile was determined by calculating the fractional inhibitory concentration index (FICI) according to the formula: FICI = (MIC of cerivastatin in combination/MIC of cerivastatin alone) + (MIC of itraconazole in combination/MIC of itraconazole alone) [22]. The drug combination effects were considered synergistic if FICI ≤ 0.5, indifferent if FICI > 0.5 and ≤4, and antagonist if FICI > 4 [23]. The most effective combinations were identified as those yielding the lowest FICI scores. All findings represent results obtained from at least three independent experiments.

### 4.6. Scanning Electron Microscopy (SEM)

To examine surface ultrastructural alterations, *S. brasiliensis* yeast cells were exposed for 48 h to 0.125 μM itraconazole and 0.15 μM cerivastatin, either individually or together. After treatment, cells were washed with phosphate-buffered saline (PBS) and chemically fixed for 1 h in a solution containing 2.5% glutaraldehyde and 4% formaldehyde prepared in 0.1 M cacodylate buffer. Samples were subsequently washed, adhered to poly-L-lysine–coated glass coverslips (Sigma-Aldrich^®^), and post-fixed for 30 min in 1% osmium tetroxide supplemented with 1.25% potassium ferrocyanide in cacodylate buffer. A graded ethanol dehydration series (30–100%, 20 min per step) was performed before critical-point drying in CO_2_. Specimens were then sputter-coated with gold and visualized using an FEI Quanta 250 scanning electron microscope (FEI Company, Hillsboro, OR, USA). Digital images were processed with Adobe Photoshop CS6 software.

### 4.7. Transmission Electron Microscopy (TEM)

For internal ultrastructural analysis, yeast cells were treated under the same drug conditions described above. After washing with PBS, fixation was carried out in 2.5% glutaraldehyde and 4% formaldehyde in 0.1 M cacodylate buffer for 48 h. Cells were washed and post-fixed in 1% osmium tetroxide containing 1.25% potassium ferrocyanide for 1 h, followed by dehydration in ascending acetone concentrations (15–100%, 20 min per step). Dehydrated samples were embedded in Spurr resin, sectioned into ultrathin slices, and contrasted with uranyl acetate. Imaging was obtained using a Hitachi HT 7800 transmission electron microscope (Hitachi High-Technologies Corporation, Tokyo, Japan), and micrographs were digitally processed using Adobe Photoshop.

### 4.8. Flow Cytometry Analysis

To assess lipid-related alterations, yeast cells subjected to the same treatment conditions described above were processed for flow cytometry. Cells were washed three times with PBS and incubated with 20 μM BODIPY 496/503 (Thermo Fisher Scientific, Waltham, MA, USA) for 30 min at room temperature in the absence of light. Following staining, cells were washed, fixed in 2% formaldehyde in PBS, and washed again. Data acquisition was conducted using a BD Accuri™ C6 flow cytometer (BD Biosciences, San Jose, CA, USA), with 10,000 events recorded per sample. Analyses were carried out using BD Accuri C6 1.0 software. Experiments were independently repeated three times. Statistical comparisons were performed using one-way ANOVA followed by Tukey’s post hoc test, adopting a significance threshold of *p* < 0.05 (GraphPad Prism 8.4, GraphPad Software Inc., San Diego, CA, USA).

## Figures and Tables

**Table 1:** Compounds from the NIH Clinical Collection library that promoted growth inhibitions greater than 80% in *Sporothrix brasiliensis*.

Compounds	Codes	Use	% Inhibition at 10 µM
Itraconazole	Reference antifungal used in the tests ^a^		91
Aripiprazole	SAM001246750	Antipsychotic	98
Bifonazole	SAM001246775	Topical antifungal	88
Cerivastatin sodium	SAM001246554	Treatment of hypercholesterolemia and prevention of cardiovascular diseases	91
Ketoconazole	SAM001246983	Topical antifungal	96
Idarubicin hydrochloride	SAM001246676	Leukemia treatment	88
Chloroxine	SAM002554895	Topical antifungal	91
Clotrimazole	SAM001247056	Topical antifungal	99
Disulfiran	SAM001247028	Alcoholism treatment	97
Econazole nitrate	SAM002554898	Topical antifungal	96
Ebselen	SAM001247071	Protects against oxidative damage	89
Epigallocatechin gallate	SAM001247031	Natural antioxidant	89
Griseofulvin	SAM002589940	Oral antifungal	81
Hexachlorophene	SAM002554903	Antiseptic with antibacterial action	97
Itraconazole	SAM001246679	Oral antifungal	81
Miconazole nitrate	SAM002264623	Topical antifungal	97
Nisoldipine	SAM001246719	Treatment of angina and hypertension	100
Oligomycin A	SAM006031219	ATP synthase inhibitor	96
Oxiconazole nitrate	SAM001246724	Topical antifungal	100
5-Nonyloxytryptamine hydrochloride	SAM001247103	Serotonergic agonist	100
Tacrolimus	SAM001246677	Immunosuppressant	86
Terbinafine hydrochloride	SAM001246565	Topical and oral antifungal	100
Toremifene citrate	SAM001246774	Breast cancer treatment	93
Triclabendazole	SAM001246681	Oral anthelmintic	98
Triclosan	SAM002554907	Antiseptic and preservative with antibacterial action	97
Trifluoperazine hydrochloride	SAM001247046	Antipsychotic	83
Voriconazole	SAM001246664	Oral antifungal	94

^a^ Itraconazole from Sigma-Aldrich^®^ (St. Louis, MO, USA) with purity greater than 98%.

**Table 2:** Minimum inhibitory concentration values of selected compounds from the NIH Clinical Collection library against *Sporothrix* spp. yeasts.

Compounds	Minimum Inhibitory Concentration ^a^ (µM)		
	*S. brasiliensis* ATCC MYA 4823	*S. schenckii* ATCC 32286	*S. globosa* CBS 130104
Itraconazole ^b^	0.25	0.5	0.125
Aripiprazole	10	5	5
Cerivastatin	1.25	1.25	0.6
Idarubicin hydrochloride	2.5	2.5	2.5
Disulfiram	5	10	5
Epigallocatechin gallate	5	>10	5
Hexachlorophene	5	10	5
Nisoldipine	10	5	5
5-Nonyloxytryptamine hydrochloride	2.5	5	2.5
Toremifene	10	10	5
Triclabendazole	10	5	5
Trifluoperazine	10	10	5

^a^ The minimum inhibitory concentration was defined as the lowest concentration of the compound that inhibited 50% or more of fungal growth. ^b^ Reference antifungal.

**Table 3:** Percentage of inhibition of statins and their structures.

Statins	Code	Launched ^a^	Structure ^b^	% Inhibition [10 µM]
Cerivastatin	SAM001246554	1998	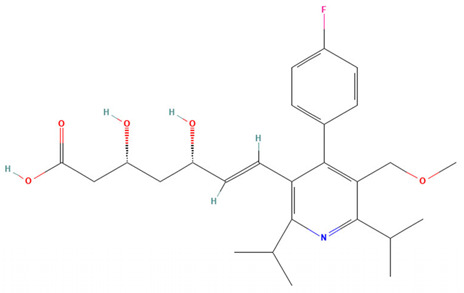	91
Simvastatin	SAM002589969	1988	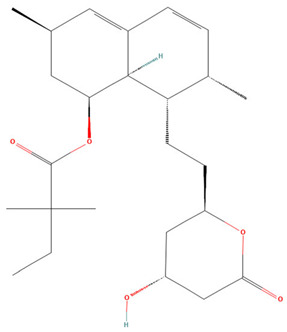	62
Fluvastatin	SAM002548940	1993	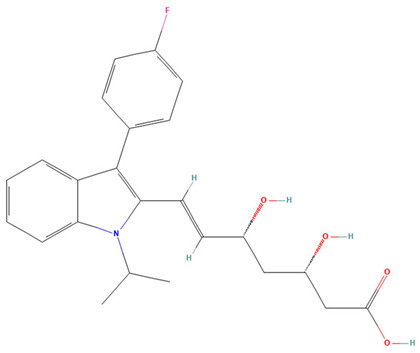	61
Lovastatin	SAM002589963	1995	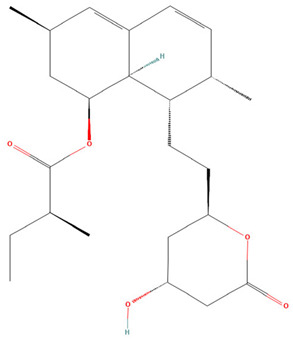	56
Itavastatin	SAM001246803	2003	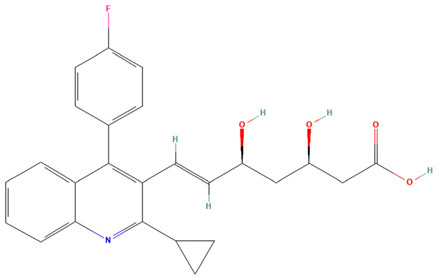	56
Mevastatin	SAM001246644	Preclinical	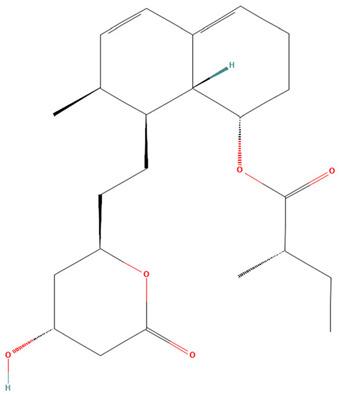	34
Pravastatin	SAM001246656	1989	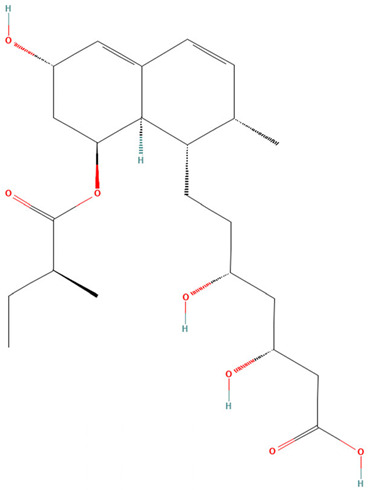	29

^a^ [9]. ^b^ Structure obtained from PubChem [10].

## Data Availability

The original contributions presented in this study are included in the article/Supplementary Materials. Further inquiries can be directed to the corresponding author.

## Supplementary Materials

The following supporting information can be downloaded at: https://www.mdpi.com/article/10.3390/ph19030479/s1, Table S1: Percentage of growth inhibition of *Sporothrix brasiliensis* after exposure to 10 µM of the library compounds for 48 h.

## References

[1] Bongomin F., Gago S., Oladele R.O., Denning D.W. (2017). Global and Multi-National Prevalence of Fungal Diseases-Estimate Precision. J. Fungi.

[2] Denning D.W. (2024). Global incidence and mortality of severe fungal disease. Lancet Infect. Dis..

[3] Rauseo A.M., Coler-Reilly A., Larson L., Spec A. (2020). Hope on the Horizon: Novel Fungal Treatments in Development. Open Forum Infect. Dis..

[4] Liu N., Wang C., Su H., Zhang W., Sheng C. (2016). Strategies in the discovery of novel antifungal scaffolds. Future Med. Chem..

[5] Rodrigues A.M., Gonçalves S.S., de Carvalho J.A., Borba-Santos L.P., Rozental S., de Camargo Z.P. (2022). Current Progress on Epidemiology, Diagnosis, and Treatment of Sporotrichosis and Their Future Trends. J. Fungi.

[6] World Health Organization (2022). WHO Fungal Priority Pathogens List to Guide Research, Development and Public Health Action.

[7] Borba-Santos L.P., Reis de Sá L.F., Ramos J.A., Rodrigues A.M., de Camargo Z.P., Rozental S., Ferreira-Pereira A. (2017). Tacrolimus Increases the Effectiveness of Itraconazole and Fluconazole against *Sporothrix* spp.. Front. Microbiol..

[8] Benelli J.L., Poester V.R., Munhoz L.S., Melo A.M., Trápaga M.R., Stevens D.A., Xavier M.O. (2021). Ebselen and diphenyl diselenide against fungal pathogens: A systematic review. Med. Mycol..

[9] Cortellis Drug Discovery Intelligence Database. https://www.cortellis.com/drugdiscovery/.

[10] PUBCHEM National Center for Biotechnology Information. https://pubchem.ncbi.nlm.nih.gov/.

[11] Gutierrez-Perez C., Cramer R.A. (2025). Targeting fungal lipid synthesis for antifungal drug development and potentiation of contemporary antifungals. npj Antimicrob. Resist..

[12] Cerivastatin. https://www.cortellis.com/drugdiscovery/entity/drug/189237.

[13] Tavakkoli A., Johnston T.P., Sahebkar A. (2020). Antifungal effects of statins. Pharmacol. Ther..

[14] Parihar S.P., Guler R., Brombacher F. (2019). Statins: A viable candidate for host-directed therapy against infectious diseases. Nat. Rev. Immunol..

[15] Sun W., Park Y., Sugui J.A., Fothergill A., Southall N., Shinn P., McKew J.C., Kwon-Chung K.J., Zheng W., Williamson P.R. (2013). Rapid identification of antifungal compounds against *Exserohilum rostratum* using high throughput drug repurposing screens. PLoS ONE.

[16] Custodio H., Norton T., Fortwendel J. (2015). Growth Inhibitory Effect of Cerivastatin Against Yeasts and *Aspergillus fumigatus*. Open Forum Infect. Dis..

[17] Abdel-Rahman S., Preuett B.L., Leeder J.L. (2021). Dermatophytosis Prophylaxis and Treatment. U.S. Patent.

[18] Zhang Q., Liu F., Zeng M., Mao Y., Song Z. (2021). Drug repurposing strategies in the development of potential antifungal agents. Appl. Microbiol. Biotechnol..

[19] Eliaš D., Tóth Hervay N., Gbelská Y. (2024). Ergosterol Biosynthesis and Regulation Impact the Antifungal Resistance and Virulence of *Candida* spp.. Stresses.

[20] BLAST Program. https://blast.ncbi.nlm.nih.gov/Blast.cgi.

[21] EUCAST-AFST (2023). Method for the Determination of Broth Dilution Minimum Inhibitory Concentrations of Antifungal Agents for Yeasts.

[22] Pillai S.K., Moellering R.C., Eliopoulos G.M. (2005). Antibiotics in Laboratory Medicine.

[23] Odds F.C. (2003). Synergy, antagonism, and what the chequerboard puts between them. J. Antimicrob. Chemother..

